# Smelling different after COVID-19 ?

**DOI:** 10.1192/j.eurpsy.2022.1309

**Published:** 2022-09-01

**Authors:** K. Nourchene, E. Khelifa, B. Abassi, I. Bouguerra, O. Maatouk, S. Ben Aissa, L. Mnif

**Affiliations:** 1 Razi hospital, Skolly, Tunis, Tunisia; 2 Razi Hospital, F Adult Psychiatry Department, Manouba, Tunisia; 3 Errazi hospital-Mannouba, F, Mannouba, Tunisia; 4 Razi, Skolly, Manouba, Tunisia

**Keywords:** Covid-19, olfactory hallucination, phantosmia

## Abstract

**Introduction:**

Over the course of COVID 19 illness, olfactory dysfunction was largely described as hyposmia and anosmia. What about phantosmia?

**Objectives:**

In this study, we aimed to explore olfactory hallucinations among COVID 19 patients.

**Methods:**

Our literature review was based on the PubMed interface and adapted for 2 databases: Science Direct and Google Scholar using the following combination ( phantosmia [MeSH terms]) OR (olfactory hallucinations[MeSH terms]) AND (COVID-19 [MeSH terms]).

**Results:**

Smell dysfonction is one of the most revealing sign of COVID 19 infection. However, other symptoms particularlty phantosmia tend to emerge later in the course of the disease. Female predominance was noted among patients sufferning from olfactory hallucinations regardless to their medical history. An unpleasant olfactory sensation was the most described sign. The occurene of phantosmia was also described in one case of women suffering from schizophrenia whom tested positive for COVID 19 infection.

**Conclusions:**

Olfactory hallucinations are more and more associated with COVID-19 disease regardless to psychiatric disorders. The pathological mechanism remains unclear and further studies are needed for a better comprehension and management.

**Disclosure:**

No significant relationships.

